# Cutavirus on the skin in an Asian cohort: identification of a novel geographically related genotype

**DOI:** 10.1186/s12985-023-02029-8

**Published:** 2023-04-17

**Authors:** Yumiko Hashida, Tomonori Higuchi, Masanori Daibata

**Affiliations:** Department of Microbiology and Infection, Kochi Medical School, Kochi University, Nankoku, Kochi 783-8505 Japan

**Keywords:** Cutavirus, Skin, Prevalence, Viral load, Persistent infection, Genetic variability

## Abstract

**Background:**

Cutavirus (CuV) is the newest human parvovirus and is currently receiving increasing attention because of its possible association with cutaneous T-cell lymphoma. Despite the pathogenetic potential of CuV, it has been detected in normal skin; however, little is known about the prevalence, infection levels, and genetic variations of this virus in the skin of the general population.

**Methods:**

We investigated the CuV DNA prevalence and viral loads concerning age, sampling location, and gender using 678 skin swabs collected from the normal-appearing skins of 339 Japanese participants aged 2–99 years. Phylogenetic analyses were also conducted based on the near-full-length CuV sequences identified in this study.

**Results:**

Both the CuV DNA prevalence and viral loads were significantly higher in the skin of elderly persons aged ≥60 years compared with those of persons aged < 60 years. CuV DNA tended to persist in the skin of elderly individuals. No significant difference in viral loads was observed between the skin of the upper arm and the skin of the forehead in CuV DNA-positive specimens. Significantly higher viral loads were evident in men vs. women, although no gender-associated differences in viral prevalence were noted. Phylogenetic analyses demonstrated the existence of Japanese-specific viruses that were genetically distinct from viruses prevalent in other areas, especially Europe.

**Conclusions:**

This large study suggests that high levels of CuV DNA are prevalent on the skin of elderly adults. Our findings also indicated the prevalence of geographically related CuV genotypes. A follow-up study of this cohort should provide helpful information on whether CuV may become pathogenic.

**Supplementary Information:**

The online version contains supplementary material available at 10.1186/s12985-023-02029-8.

## Introduction

Cutavirus (CuV), a member of the *Protoparvovirus* genus, is a small nonenveloped virus with a single-stranded linear DNA genome of approximately 4.5 kb [1–3]. This virus is the newest human parvoviruses, discovered initially in fecal specimens from Brazilian children with diarrhea using metagenomics [1]. The CuV genome was subsequently identified in cutaneous T-cell lymphoma (CTCL) using in silico analysis in preexisting metagenomic libraries; moreover, it was detected in 4/17 (24%) specimens of CTCL from French patients by polymerase chain reaction (PCR) [1]. CuV DNA was also found in 1/10 (10%) biopsy specimens from Danish patients with cutaneous melanoma [4]. Subsequent studies reported the detection of CuV DNA in 6/117 (5%) and 4/25 (16%) biopsy specimens from patients with CTCL from Germany [5] and Finland [6], respectively. Thus, studies on CuV infection have mainly focused on malignant cutaneous tumors, with a possible association between CuV and CTCL.

To date, only a few studies have assessed the prevalence of CuV in the skin of healthy individuals. No viral DNA was found in the skin biopsy specimens from three French and 98 Finnish healthy adults [1, 6]. Using skin surface swabs, a study reported the detection rate of CuV DNA (3.8%, 9/237) in the skin of immunocompetent adults in Germany [7]. Thus, despite the skin tropism and the pathogenetic potential of CuV, the prevalence and viral loads of CuV in the skin among the general population have not been well delineated. Furthermore, epidemiological studies of CuV in the skin have all stemmed from individuals living in Europe. No data are available regarding the prevalence of CuV in the skin in other areas. Therefore, a worldwide survey including an Asian population will provide useful information on the geographical distribution of this novel human virus.

Previous studies have demonstrated that some skin-tropic viruses have distinct genotypes linked to the geographic origin of the infected individuals. Merkel cell polyomavirus (MCPyV) is etiologically linked to cutaneous Merkel cell carcinoma (MCC) [8]; moreover, it exists in healthy skin as a skin commensal [9, 10]. Several studies have shown that MCPyV has two major geographically related genotypes, namely European/North American and Asian/Japanese genotypes [11–14]. In turn, human polyomavirus 6 (HPyV6) and human polyomavirus 7 (HPyV7) are cutaneous viruses that are comprised of the human skin virome [15, 16]. We have also shown that the viral sequences of HPyV6 and HPyV7 vary between the prevalence in the skin of white and Asian populations [17]. Because it has been suggested that CuV may be part of the human skin virome [2], these findings highlight the need for studies to determine whether the CuV strains present in the skin also exhibit genotypic variation between white and Asian populations.

Based on this background, we were the first to investigate the age-specific prevalence and viral loads of CuV in 678 swab specimens collected from two different areas of the normal-appearing skin of individuals in a large Asian cohort. We also estimated the persistence and acquisition of CuV DNA in a subset of this cohort. Moreover, we conducted the first phylogenetic analyses of normal-skin-derived CuV strains using the near-full-length CuV genome, revealing the existence of Japanese-specific viruses genetically distinct from previously reported strains from Europe.

## Materials and methods

### Study participants and specimen collection

This study cohort included 339 Japanese individuals (persons of Japanese descent who resided in Japan) enrolled from 2014 to 2020. The participants ranged from 2 to 99 years (median age, 69 years); 213 were women, and 126 were men. In this study, gender refers to socially constructed roles, behaviors, and identities of women, men, and gender-diverse people, according to the Sex and Gender Equity in Research (SAGER) guidelines [18]. The authors interviewed the participants in this study for personal information, including current medical history of cancer and skin diseases and medication history. The participants and their families declared they were free of cancer, skin diseases, or having received immunosuppressive agents. The local skin areas from which the swabs were collected were carefully inspected and confirmed to appear healthy. However, the rest of the skin was not further examined. A total of 678 skin smear specimens were collected from about 50 cm^2^ of normal-appearing skin in two different regions in the same individuals, including the skin of the upper arm and the skin from the forehead to the top of the head. Sample collection was performed by rubbing the skin back and forth 5–10 times using sterile cotton swabs moistened with phosphate-buffered saline (PBS). All swabs were placed in a separate vial containing sterile PBS, and DNA was extracted within 2 h of sample collection. The subjects were divided according to age into six groups (Table 1).

**Table 1:** Age-specific prevalence of cutavirus DNA in swab specimens of normal skin of the arm and forehead

Subject Age, y	Tested, No.		Arm skin specimens		Forehead skin specimens		Arm or forehead skin specimens^a^		Arm and forehead skin specimens^b^
			Positive for CuV, No. (%)		Positive for CuV, No. (%)		Positive for CuV, No. (%)		Positive for CuV, No. (%)
2–19	27		2 (7)		2 (7)		0 (0)		2 (7)
20–39	59		1 (2)		2 (3)		1 (2)		1 (2)
40–59	53		8 (15)		6 (11)		10 (19)		2 (4)
60–79	68		12 (18)		9 (13)		11 (16)		5 (7)
80–89	68		17 (25)		28 (41)		23 (34)		11 (16)
90–99	64		13 (20)		27 (42)		20 (31)		10 (16)
Total	339		53 (15.6)		74 (21.8)		65 (19.2)		31 (9.1)

*CuV* cutavirus, *y* year

^a^ viral detection only in one of the two skin areas

^b^ viral detection in both skin areas

### Quantification of the viral genome

DNA extraction was performed using the standard phenol: chloroform method. Concentrations of extracted DNAs were measured using BioPhotometer Plus (Eppendorf, Hamburg, Germany). Twenty-nanogram aliquots of the extracted DNA were analyzed for the detection and quantification of CuV using TaqMan-based real-time quantitative PCR (qPCR), as described elsewhere [19]. Primers and probes were prepared to amplify a region of the gene encoding the CuV viral capsid protein 2 (*VP2*). The forward and reverse primers are located at nucleotide positions 4245–4269 and 4316–4335 based on the GenBank CuV sequence [accession number NC_039050]), respectively (Additional file 1: Table S1) [6]. The reaction mixture was prepared in a total volume of 20 µl containing the TaqMan Gene Expression Master Mix (Thermo Fisher Scientific, Waltham, MA, USA), 900 nM of each primer, and 250 nM dual-labeled probe. Water was added to the PCR reaction mixture instead of extracted DNA as a negative PCR-amplification control in each PCR run. The negative control did not show any amplification in all of the specimens tested in this study. Precautions were also taken to prevent contamination in the PCR assays as described elsewhere [20], although negative DNA extraction controls were not included. The *RNase P* gene was amplified in separate PCR runs as a positive control to confirm the presence of PCR-amplifiable DNA. A PCR assay was performed using the same primers, and the PCR product (CuV *VP2* sequence; nucleotide positions 4245–4335) was cloned into the pMD20-T vector (Takara Bio, Shiga, Japan). We prepared 10-fold serial dilutions using the cloned plasmid DNA to generate a standard curve, from which we calculated the viral copy number. The results are expressed as viral DNA copies/ng DNA. Because samples with cycle threshold values ≤ 41 were considered positive for targeted viral nucleic acids [13], we considered swab specimens with ≥ 3 × 10^–2^ copies/ng DNA to be positive.

### Viral DNA sequencing analysis

The near-full-length CuV sequences (4455 bp, nucleotide positions 2–4456) were amplified by PCR using different combinations of 13 primer sets (Additional file 1: Table S1). The purified PCR products were sequenced directly. A total of 36 CuV sequences obtained in this study were deposited in the GenBank database under accession numbers LC744018–LC744023 and LC760811–LC760840.

### Phylogenetic analysis and nucleotide identity analysis

The nucleotide sequences were aligned using Clustal W [21]. Phylogenetic trees were constructed using the maximum-likelihood method in MEGA X [22]. The bootstrap values were based on 1000 replicates for all trees. Nucleotide identity analysis was performed using the nucleotide BLAST program with default parameters [23].

### Statistical analyses

Any correlations with CuV positivity rates were analyzed using Fisher’s exact test or Pearson’s chi-squared test if any values were < 5. The differences in viral loads were compared using the Mann–Whitney nonparametric *U* test. All statistical analyses were performed using R, version 1.61, with its graphical user interface, EZR [24]. Significance was set at *P* < 0.05.

## Results

### CuV DNA prevalence and viral loads in normal skin

We tested 678 swab specimens obtained from the normal-appearing skin of the upper arm and forehead of 339 Japanese individuals, to detect and quantify the CuV genome. All specimens were tested twice independently in a blind manner. The results of the first and second tests were concordant for CuV DNA positivity or negativity in 657 of 678 (96.9%) specimens. The remaining samples with low CuV loads were all around the 3 × 10^–2^ copies/ng DNA borderlines and were determined to be positive or negative on the third test.

Overall, CuV DNA was detected in 53 of 339 (15.6%) arm skin specimens and 74 of 339 (21.8%) forehead skin specimens. The age-related prevalence was assessed. The detection rate according to the swab-sampling site in each age group is listed in Table 1. The positivity rates in groups aged 2–59 years were 7.9% (11/139) for the arm swabs and 7.2% (10/139) for the forehead swabs; the respective rate significantly increased to 21.0% (42/200; *P* = 0.002) and 32.0% (64/200; *P* < 0.001) in older groups (individuals aged 60–99 years). The rates of simultaneous detection of CuV DNA in both the arm and forehead skin specimens in the same individuals were also significantly higher in groups aged 60–99 years (13.0% [26/200]) than they were in younger groups (3.6% [5/139]; *P* = 0.003). No significant difference in site-specific detection rates between the arm and forehead was observed among the age groups (*P* = 0.354). The detection rate on either of the skins according to gender in each age group is listed in Table 2. Overall, no gender-associated differences in CuV DNA prevalence were noted (29.6% [63/213] for women and 26.2% [33/126] for men; *P* = 0.586).

**Table 2:** Gender-specific prevalence of cutavirus DNA in skin swab specimens according to age group

Subject Age, y	Specimens from women			Specimens from men	
	Tested, No.	Positive for CuV, No. (%)^a^		Tested, No.	Positive for CuV, No. (%)^a^
2–19	21	1 (5)		6	1 (17)
20–39	27	0 (0)		32	2 (6)
40–59	27	5 (19)		26	7 (27)
60–79	34	8 (24)		34	8 (24)
80–89	47	22 (47)		21	12 (57)
90–99	57	27 (47)		7	3 (43)
Total	213	63 (29.6)		126	33 (26.2)

*CuV* cutavirus, *y* year

^a^ Viral detection in either the arm or forehead specimens

**Fig. 1:**
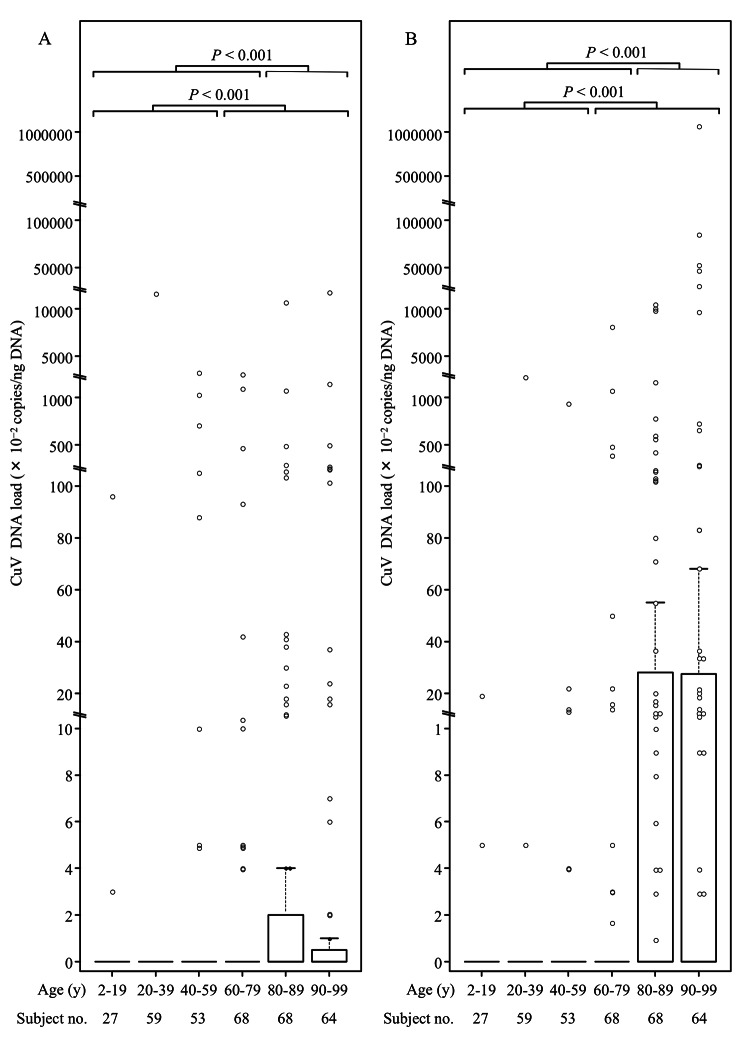
Box plots showing age-specific cutavirus (CuV) DNA loads in skin swabs. (A) CuV DNA loads in skin swabs from the upper arm. (B) CuV DNA loads in skin swabs from the forehead. The horizontal bars extend the median by 1.5 times the interquartile ranges. The comparisons of the viral loads between age groups were performed using the Mann–Whitney nonparametric *U* test, and *P* values are presented above the box plots. The number of subjects is presented for each age group below the box plots

**Fig. 2:**
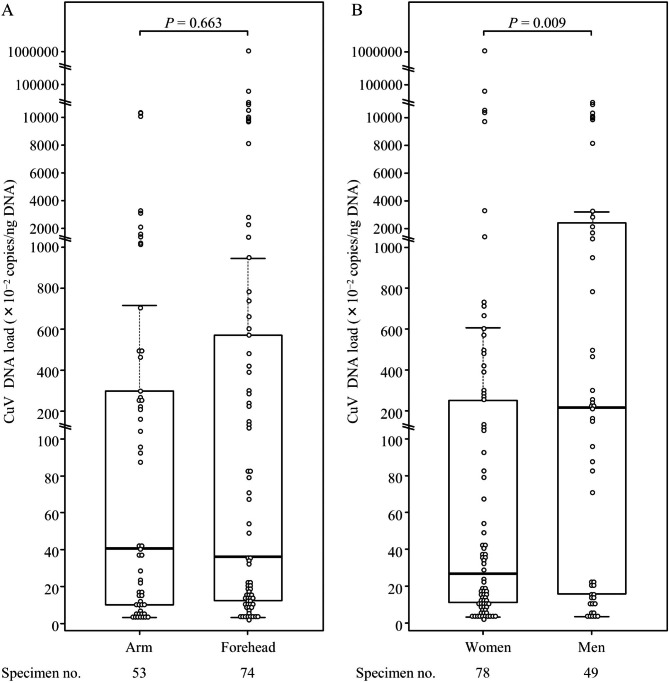
Box plots showing cutavirus (CuV) DNA loads in skin swabs positive for CuV DNA. (A) CuV DNA loads according to swab-sampling location. (B) CuV DNA loads according to gender. The horizontal bars extend the median by 1.5 times the interquartile ranges. The comparisons of the viral loads between age groups were performed using the Mann–Whitney nonparametric *U* test, and *P* values are presented above the box plots. The number of specimens is presented for each group below the box plots

Next, we evaluated the CuV loads in skin swabs according to age group. Box plots of CuV DNA levels showed significantly higher viral loads in both the arm and forehead skins of persons aged ≥60 years compared with those of persons aged < 60 years (*P* < 0.001; Fig. 1). Thus, high loads of CuV DNA likely to exist in the skin of elderly individuals. For example, a CuV load of > 100 copies/ng DNA was detected in the arm and/or forehead skin in nine subjects; eight of whom (89%) were older than 80 years. Of note, a 91-year-old woman carried a very high CuV load of > 10,000 copies/ng DNA in her forehead skin without any apparent symptoms. We also conducted comparative analyses of the CuV loads between skin swabs obtained from the arms and foreheads and between those from women and men in CuV DNA-positive specimens (Fig. 2). No significant difference in site-specific viral loads was detected between the arm and forehead samples (*P* = 0.663). In turn, significantly higher viral loads were evident in men vs. women (*P* = 0.009).

### Persistence and acquisition of CuV DNA in normal skin

We reassessed 46 subjects aged 32–98 years from whom both arm and forehead skin swabs were obtained approximately 6 months after collecting the first sample. The second specimens were collected from the same area as the first specimens, albeit with some minor variation in the exact site of collection. To investigate viral persistence, defined as the presence of CuV DNA at the two consecutive visits, we tested five and nine subjects whose first arm and forehead swabs contained viral DNA, respectively (Table 3). Of these individuals, three (60%) and four (44%), respectively, were found to have retained the CuV genome at a comparable level in the second skin swabs. Of note, all individuals with retained CuV DNA were older than 70. To investigate viral acquisition, defined as the new detection of CuV DNA that was not detected at the first visit, we tested 41 and 37 subjects whose first arm and forehead swabs were negative for CuV DNA, respectively (Table 3). The CuV viral genome was detected in the second skin swabs of five (12%) and 10 (27%) of these individuals, respectively. Similar to that observed for viral persistence, the acquisition of the CuV genome tended to occur more frequently in the skin of elderly individuals.

**Table 3:** Persistence and acquisition of cutavirus DNA among individuals in Japan

All subjects^a^									Subjects aged 60 ≥ years					
Arm skin specimens				Forehead skin specimens					Arm skin specimens				Forehead skin specimens	
Tested, No.	Positive for CuV, No. (%)			Tested, No.		Positive for CuV, No. (%)			Tested, No.	Positive for CuV, No. (%)			Tested, No.	Positive for CuV, No. (%)
Persistence^b^														
5	3 (60)			9		4 (44)			3	3 (100)			8	4 (50)
Acquisition^c^														
41	5 (12)			37		10 (27)			34	5 (15)			29	9 (31)
Subjects with viral persistence														
Specimens,							Measurement (× 10^–2^ copies/ng DNA)							
Subject No.		Age, y			Gender		First specimens				Second specimens^d^			
Arm skin														
130		83			W		1482				204			
170		92			W		7				6			
192		72			M		10				59			
Forehead skin														
32		90			M		83				25			
104		94			W		9				11			
112		79			W		16				38			
130		83			W		606				4521			
Subjects with viral acquisition														
Specimens,							Measurement (× 10^–2^ copies/ng DNA)							
Subject No.		Age, y			Gender		Second specimens^e^							
Arm skin														
32		90			M		6							
67		98			W		17							
98		90			W		29							
102		90			W		5							
183		68			W		10							
Forehead skin														
67		98			W		124							
71		75			W		15							
88		79			W		4							
93		93			W		34							
95		95			W		23							
102		90			W		6							
103		84			W		5							
128		73			M		4							
163		44			M		8							
184		71			W		6							

*CuV* cutavirus, *M* man, *W* woman, *y* year

^a^ A total of 46 subjects could be tested for CuV DNA in the second specimens

^b^ Defined as the presence of CuV DNA at the two consecutive visits with an interval of approximately 6 months

^c^ Defined as the new detection of CuV DNA that was not detected at the first visit

^d^ Second specimens were obtained from subjects who tested positive for CuV DNA in the first specimens and measured for viral copy number after an interval of approximately 6 months

^e^ Second specimens were obtained from subjects who tested negative for CuV DNA in the first specimens and measured for viral copy number after an interval of approximately 6 months

### Phylogenetic analysis and geographic genetic diversity of CuV

**Fig. 3:**
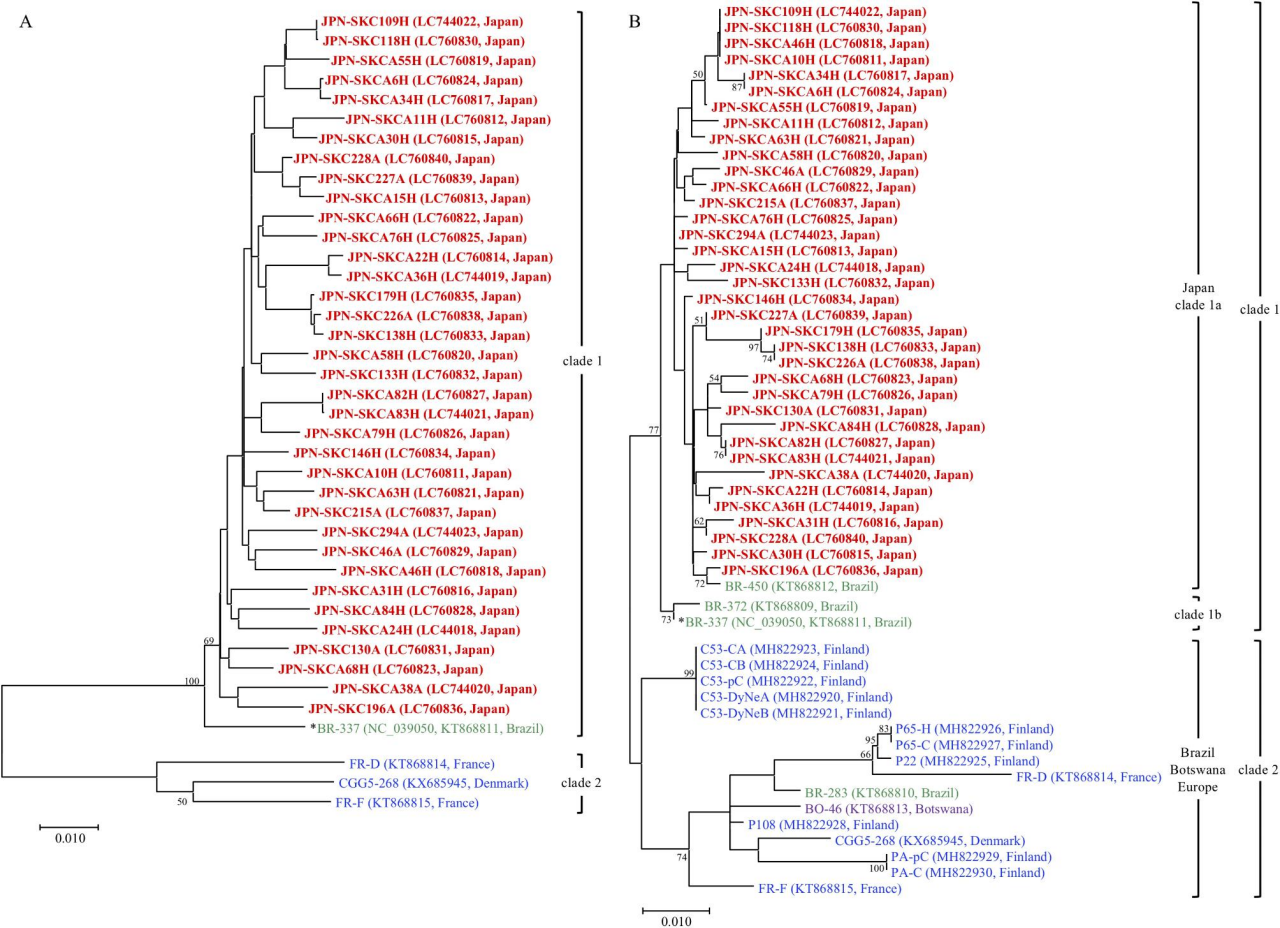
Phylogenetic trees generated using the maximum-likelihood method. (A) A phylogenetic tree was constructed based on 40 near-full-length cutavirus (CuV) sequences (4455 bp). These included 36 sequences that were successfully amplified from skin swab specimens from the Japanese individuals included in this study and four sequences retrieved from GenBank. The two major nucleotype clades (clades 1 and 2) are indicated. (B) A phylogenetic tree was constructed based on 55 CuV viral capsid protein 1 (*VP1*)/*VP2* sequences with 538 bp (nucleotide positions 3778–4315 bp). These included the 36 sequences from the Japanese individuals and 19 sequences recovered from individuals of various geographic origins that were retrieved from GenBank as of December 1, 2022. The CuV reference sequence of the BR-337 strain (GenBank accession number no., NC_039050) is indicated with an asterisk. The sequences obtained from the Japanese individuals in the present study are presented in bold and colored in red. The CuV strains from Brazil, Botswana, and Europe are colored green, purple, and blue, respectively. The Brazilian and Botswanan strains were from fecal specimens, whereas the remaining strains were from skin specimens. The names of the sequences are indicated together with the GenBank accession numbers and country of origin in parentheses. The percentage bootstrap values calculated from 1000 replicates are indicated at the internal nodes. The scale bars represent the number of substitutions per site

Next, we analyzed the phylogenetic relationships between the CuV recovered from skin specimens in the Japanese population studied in this study and those reported previously in other populations. In this study, 36 near-full-length CuV sequences (4455 bp) were obtained successfully from the skin swabs of different individuals (nine sequences from the arm and 27 from the forehead), irrespective of the viral load of the specimens and the age/gender of the subjects (Table 4). As of December 1, 2022, four near-complete CuV sequences have been deposited in GenBank. These include sequences from one virus, termed BR-337, that was first identified in a fecal specimen from a patient in Brazil (GenBank accession no. KT868811) [1]; two viruses, termed FR-D and FR-F, that were recovered from skin biopsy specimens from patients with CTCL from France (KT868114 and KT868815, respectively) [1]; and one virus, termed CGC5-268, from a melanoma sample from a patient from Denmark (KX685945) [4]. We performed a phylogenetic study using these 40 CuV sequences (Fig. 3A). The phylogenetic tree generated using the maximum-likelihood method showed that all of the Japanese CuV sequences formed one major clade (designated clade 1) that included the Brazilian BR-337 sequence but was clearly distinct from a clade (clade 2) comprising an additional three sequences originating from European individuals. Slight sequence differences were seen in the phylogenetic tree of the 36 Japanese CuV sequences amplified from distinct individuals.

**Table 4:** Nucleotide identities between the near-full-length cutavirus sequences (4455 bp) identified in the current and previous studies

Subject		Viral load × 10^–2^ copies/ng DNA	Strain	% Nucleotide identity			
Age	Gender			BR-337 [NC_039050]^a^	FR-D [KT868814]	FR-F [KT868815]	CGG5-268 [KX685945]
83	M	12,227	JPN-SKCA6H [LC760824]	97.67	93.88	94.44	93.99
94	W	9757	JPN-SKCA10H [LC760811]	97.69	93.90	94.53	94.03
99	W	734	JPN-SKCA11H [LC760812]	97.76	93.88	94.13	93.90
89	M	243	JPN-SKCA15H [LC760813]	97.96	93.93	94.37	94.21
88	M	158	JPN-SKCA22H [LC760814]	97.55	94.13	94.46	94.10
87	M	10,122	JPN-SKCA24H [LC744018]	97.44	94.06	94.44	94.26
87	W	431	JPN-SKCA30H [LC760815]	98.00	94.15	94.37	94.08
91	M	47,879	JPN-SKCA31H [LC760816]	97.60	93.84	94.37	94.21
93	W	83	JPN-SKCA34H [LC760817]	97.73	93.93	94.41	93.94
75	W	491	JPN-SKCA36H [LC744019]	97.49	94.04	94.42	94.03
94	W	280	JPN-SKCA38A [LC744020]	97.42	93.86	94.55	94.15
96	W	31,584	JPN-SKCA46H [LC760818]	97.58	93.99	94.71	94.30
72	M	8196	JPN-SKCA55H [LC760819]	97.67	94.10	94.46	94.08
93	W	85,864	JPN-SKCA58H [LC760820]	97.62	93.95	94.30	94.05
84	M	2361	JPN-SKCA63H [LC760821]	97.64	93.81	94.37	94.15
83	W	80	JPN-SKCA66H [LC760822]	97.67	93.97	94.44	94.12
85	M	71	JPN-SKCA68H [LC760823]	97.87	94.11	94.66	94.24
96	W	668	JPN-SKCA76H [LC760825]	97.55	93.92	94.39	94.12
88	M	9898	JPN-SKCA79H [LC760826]	97.73	94.04	94.39	94.08
80	W	570	JPN-SKCA82H [LC760827]	97.91	94.13	94.70	94.24
91	W	1,068,809	JPN-SKCA83H [LC744021]	97.87	94.08	94.66	94.19
95	F	287	JPN-SKCA84H [LC760828]	97.40	93.86	94.23	93.94
19	M	96	JPN-SKC46A [LC760829]	97.58	93.81	94.42	93.99
92	M	53,678	JPN-SKC109H [LC744022]	97.69	94.06	94.50	94.10
49	M	944	JPN-SKC118H [LC760830]	97.67	94.04	94.48	94.08
83	W	1482	JPN-SKC130A [LC760831]	97.85	93.95	94.55	94.21
86	W	124	JPN-SKC133H [LC760832]	97.73	93.90	94.48	93.92
86	M	228	JPN-SKC138H [LC760833]	97.80	93.97	94.53	94.10
29	M	2874	JPN-SKC146H [LC760834]	97.78	93.84	94.35	93.96
82	M	787	JPN-SKC179H [LC760835]	97.85	93.95	94.53	94.06
63	W	93	JPN-SKC196A [LC760836]	97.60	93.99	94.35	93.92
46	M	215	JPN-SKC215A [LC760837]	97.91	94.17	94.64	94.39
40	M	88	JPN-SKC226A [LC760838]	97.73	93.93	94.44	94.06
44	W	715	JPN-SKC227A [LC760839]	97.82	93.95	94.37	94.24
42	M	1030	JPN-SKC228A [LC760840]	97.67	93.77	94.26	93.99
75	M	1452	JPN-SKC294A [LC744023]	97.64	94.02	94.46	94.19

^a^ GenBank accession numbers are indicated in square brackets

*M* man; *W* woman

We also conducted a phylogenetic study using 55 partial *VP1*/*VP2* sequences (Fig. 3B). According to the data available on GenBank on December 1, 2022, a total of 19 partial sequences of CuV *VP1*/*VP2* with 538 bp (nucleotide positions 3778–4315) have been reported from Brazil, Botswana, and Europe (including Finland, Denmark, and France), which were identified from skin or fecal specimens. As expected, all of the Japanese sequences belonged to clade 1. Importantly, the Japanese strains formed a paraphyletic group (tentatively designated Japan clade or clade 1a) distinct from other clades comprising the sequences originating from other regions (clades 1b and 2). As an exception, one strain from Brazil (BR-450, KT868812) was included in clade 1a. Thus, these phylogenetic analyses provided evidence of a specific Japanese geographic genotype of CuV. A phylogenetic tree constructed based on 44 complete CuV *VP2* sequences (including the 36 Japanese sequences and eight sequences retrieved from GenBank), together with the *VP2* sequences of gray fox amdovirus and bufavirus used as outgroups, also showed the presence of the Japan clade in the CuV group (Additional file 2: Fig. S1).

**Fig. 4:**
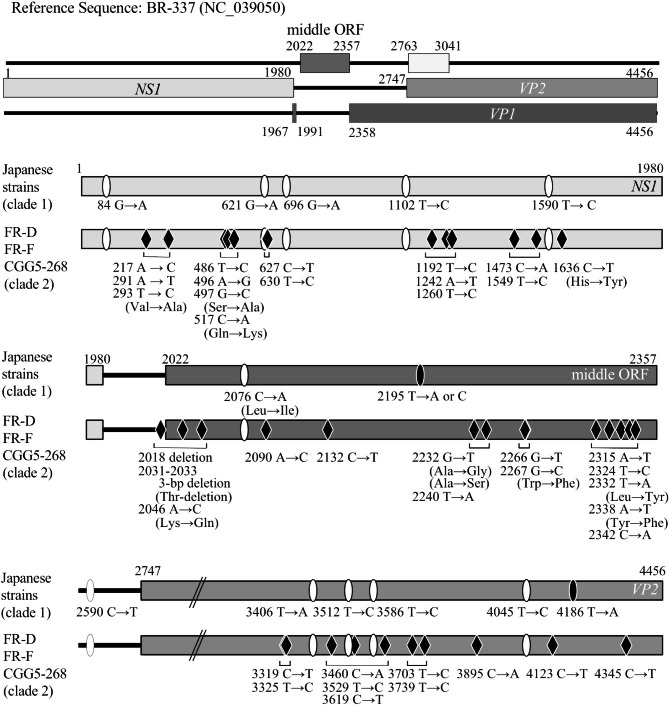
Schematic diagrams showing the summaries of cutavirus (CuV) gene alterations. The sequences of the genes encoding the nonstructural protein 1 (*NS1*) and viral capsid protein 2 (*VP2*) and the sequence of the middle open reading frame (ORF) were compared with the CuV reference sequence of the BR-337 virus. The Japanese viruses included the 36 CuV sequences obtained in this study. The FR-D, FR-F, and CGG5-268 European CuV, which belong to clade 2, were also included. Filled ovals indicate alterations specific to the Japanese viruses. Open ovals indicate alterations common to the Japanese and European viruses. Filled rhombuses indicate alterations specific to the European viruses. Amino acid replacements are also shown. Nucleotide numbers refer to the sequences of the BR-337 strain (GenBank accession number no., NC_039050).

We compared the sequences of the Japanese strains with the CuV reference sequence of BR-337 in the genes encoding the nonstructural protein 1 (*NS1*) (nucleotide positions 1–1980), the middle open reading frame (ORF) (nucleotide positions 2022–2357), and the *VP2* gene (nucleotide positions 2747–4456) which includes a part of the *VP1* gene (Fig. 4). All of the Japanese strains had the following single-nucleotide substitutions: G→A at nucleotide position 84, G→A at position 621, G→A at position 696, T→C at position 1102, and T→C at position 1590 in *NS1*; C→A at position 2076 and T→A or C at position 2195 in middle ORF; and T→A at position 3406, T→C at position 3512, T→C at position 3586, T→C at position 4045, and T→A at position 4186 in *VP2*. Compared with the Japanese strains, the European FR-D, FR-F, and CGG5-268 strains, which belonged to clade 2, exhibited many different genetic alterations, including single-nucleotide substitutions with amino acid replacements and base deletions. These alterations appear to define the characteristic phylogenetic features of the Japanese strains.

We also analyzed nucleotide identities of the near-full-length CuV sequences (4455 bp) between the 36 Japanese strains obtained in this study and four viruses, BR-337, FR-D, FR-F, and CGG5-268 (Table 4). We confirmed that our sequences of clade 1a were closer to the BR-337 sequence of clade 1b than the other sequences of clade 2. The Japanese CuV sequences were 97.40–98.00% and 93.77–94.71%, identical to the sequence of BR-337 and the sequences of FR-D, FR-F, and CGG5-268, respectively. The percent nucleotide identity between the Japanese CuV sequences was 97.53–99.98% (Additional file 3: Table S2).

## Discussion

CuV is receiving increasing attention because studies have suggested a causative role for this novel skin-tropic virus in CTCL [1, 2, 6, 25], although this warrants confirmation [5]. In this study, we examined the prevalence and viral load levels of CuV in the skin of 339 asymptomatic Japanese subjects who were free of skin diseases.

The present study had several strengths. One advantage was the availability of a large number of samples from participants. Because of the pathogenetic potential of CuV on the human skin, our survey was significant because it is thought that the presence of a virus at the site of disease development is essential for virus-associated disorders. Here, we provided the first age-specific analysis of the prevalence and quantification of CuV DNA in these skin samples. It should be noted that high burdens of CuV DNA were observed in persons aged ≥60 years. After 60 years, the viral prevalence and loads continued to increase, peaking in the groups aged ≥80 years. Very high viral loads of > 100 copies/ng DNA were predominantly observed in the skin specimens from individuals aged > 80 years. Thus, our assessment showed that the prevalence and viral loads of CuV increased with age. This may indicate a reduction of immunity with increasing age, which could lead to the activation of the virus in older individuals. In previous surveys, Väisänen et al. [6] did not detect CuV DNA among 159 skin biopsy specimens from 98 healthy immunocompetent adults (median age, 43 years; range, 18–67 years) from Finland, whereas they found it in four (2.9%) of 136 skin biopsy specimens from organ transplant recipients (median age, 62 years; range, 22–83 years), suggesting that CuV DNA is found more frequently in immunosuppressed patients. However, their finding related to healthy adults contrasts with our results. This might be attributed to the different approaches to sampling specimens because the viral detection rates obtained using surface skin swabs and dermis biopsies may vary [26]. Otherwise, because their survey of healthy adults was conducted on relatively younger individuals, CuV DNA could hardly be detected in their cohort. Wieland et al. [7] found CuV DNA more frequently in the skin swabs of human immunodeficiency virus-positive men (17.1%) than in those of healthy controls (3.8%) in Germany. Mohanraj et al. [27] analyzed fecal specimens in cohorts of patients with gastroenteritis in Finland. They showed that the CuV DNA prevalence among individuals older than 60 years (5.1%) was significantly higher than that among individuals younger than below 60 (0.2%). These findings support our observation that CuV DNA was more frequent on the skin of elderly individuals with waning immunity.

We also present the first data on the prevalence levels of the CuV genome in two different skin regions, including the skin of the forehead and the skin of the upper arm. There were no significant differences in the site-specific detection rates or viral loads, which was in contrast to the observation that the viral loads of MCPyV were higher on the forehead skin than on the arm skin [13]. In fact, MCPyV-associated MCC preferentially develops with a background of high viral load in sun-exposed skin areas such as forehead skin [10]. In contrast, mycosis fungoides, the most prevalent form of CTCL, often develop on the trunk, which is not commonly exposed to the sun [28]; however, other areas, such as the head and neck, are also affected [29, 30]. Risk factors for mycosis fungoides include older age and male sex [30, 31]. In this study, we evaluated the effect of gender differences on CuV DNA levels and found significantly higher viral loads in men. The CuV DNA prevalence patterns observed in this study should provide useful information to understand the CuV status on the skin.

We also evaluated the 6-month risk of CuV DNA persistence and acquisition. Our study showed that elderly subjects tended to retain CuV DNA in primarily infected regions. Similarly, the viral acquisition was more likely to occur in elderly individuals. These findings support the notion that the waning of the immune system with increasing age prevents the host from eradicating infecting viruses after the initial infection and/or preventing new infections. One limitation of the assay included the relatively small number of subjects for whom skin swabs were available for consecutive collection. Another limitation was that the short follow-up period of 6 months may not have allowed adequate assessment of viral persistence and may have underestimated viral acquisition. The classical human parvovirus B19 (B19V) can persist in the skin not only in symptomatic but also in healthy individuals [32–35]. Therefore, it is possible that CuV also persists after the initial infection in the skin of individuals, especially elderly persons.

Finally, this was the first report of the near-full-length CuV sequences identified in an Asian cohort. Our phylogenetic analyses demonstrated the existence of Japanese-specific CuV that were genetically distinct from the viruses recovered from fecal specimens of individuals from Brazil and Botswana and skin specimens of individuals from Europe. Based on the results of the phylogenetic analyses, we propose two major distinct genotypes, tentatively designated CuV genotypes 1 and 2 (Fig. 3). CuV genotype 1 can be divided into two subtypes; subtype 1a is predominantly composed of Japanese viruses that formed a paraphyletic clade, whereas subtype 1b includes a small number of viruses reported from other areas. CuV genotype 2 includes the majority of European viruses reported to date. Studies have shown that B19V is classified into three distinct genotypes (1, 2, and 3) and that the frequencies of the B19V genotypes vary in different continents [36–38]. Our findings suggest that different CuV genotypes are also prevalent worldwide. However, the global distribution patterns of CuV on normal skin have not been clarified; the information on CuV gene sequences available to date stems mainly from Europe, whereas that of North and South America, Africa, Oceania, and Asia is very limited or not available. Therefore, the findings should stimulate worldwide studies on geographically related variant CuV genotypes. Moreover, an important issue is whether a specific genotype is preferentially related to CuV-associated disorders. CTCL appears to be more frequent in Asian populations than in European populations [39]. In this context, whether the Japanese-specific CuV genotype represents a potential trigger of certain types of CTCL or merely indicates the presence of a Japan-related topotype warrant further investigation.

## Conclusions

We have shown in this large study that both the CuV DNA prevalence rates and viral loads in the skin were increased among elderly individuals. Hence, a prospective follow-up study of this cohort should provide useful information on whether CuV might become pathogenic. We also detected Japanese-specific CuV strains. Our findings suggest that the viral genotype may vary according to ethnicity; therefore, further analyses of specimens from different countries are warranted to strengthen our original CuV genotype classification. Finally, our findings are expected to promote studies on whether specific geographically related CuV genotypes might predispose the development of CuV infection into CTCL.

## Electronic supplementary material

Below is the link to the electronic supplementary material.

Additional file 1: table S1 Sequences of primers used for PCR analysis.

Additional file 2: figure S1 Phylogenetic analysis of cutavirus based on complete *VP2* sequences.

Additional file 3: table S2 Nucleotide identities between the near-full-length cutavirus sequences (4455 bp) identified in the current study.

## Data Availability

The data that support the findings of this study are available from the corresponding author upon reasonable request.
