# Supplementary material for: Cutavirus on the skin in an Asian cohort: identification of a novel geographically related genotype
Source: Virol J. 2023 Apr 17;20:69. doi: 10.1186/s12985-023-02029-8 (PMC10111705; doi:10.1186/s12985-023-02029-8)
Supplement: Supplementary file 1 — Additional file 1: table S1 Sequences of primers used for PCR analysis. [file 12985_2023_2029_MOESM1_ESM.pdf]

Table S1 Sequences of primers used for PCR analysis

Target	Primer Name	Sequence (5′→3′)	Amplicon Size (bp)	Nucleotide Position[a]	Reference
Real-Time PCR					
Cutavirus	F	TAACACATCCCAGAATYGTCACATA	91	4245−4335	[1]
	R	TTCCATTGTCTTGGAGTGCG			
	Probe	[FAM]AGTTKTCCTGACCACCAGAAGGTTCCA [TAMRA]			
RNase P	F	AGATTTGGACCTGCGAGCG	65	90872001−90872065	[2]
	R	GAGCGGCTGTCTCCACAAGT			
	Pprobe	[FAM]TTCTGACCTGAAGGCTCTGCGCG[TAMRA]			
Gene Sequence Analysis					
Cutavirus	CUV1F	TGGCTCTCAGCAAAGAGATGA	474	2−475	
	CUV1R	GGCCCCATTTTTCTGCAAAGTAT			
	CUV2F	CCACAGTGGCAAATGGATGG	567	429−995	
	CUV2R	GCTATGCAGGCTTGTTCACC			
	CUV3F	GCTTGCAGGCCCACATAGTA	287	926−1212	
	CUV3R	GGATGCTTGGTGATCCTGACA			
	CUV4F	ACCATGCAATAATGTGCTGCT	542	1139−1680	
	CUV4R	AGGCGTGGTTTCCCATCTTT			
	CUV5F	GCGGCCAAGCAATCAGACTA	590	1385−1974	
	CUV5R	GCTGGCATTCACATCCGTTC			
	CUV6F	GCAACAGGATATGGCCAACC	520	1924−2443	
	CUV6R	TGCAGCGTTGTCTGATGGAT			
	CUV7F	CCAACTAATCCATCAGACAACGC	419	2417−2835	
	CUV7R	CCACCTCCACCTCCTCCAAT			
	CUV8F	AACCAGGCACCAAAAGAGGT	223	2682−2904	
	CUV8R	TCACCATGATAAATAAACTCAGTCCT			
	CUV9F	GGGCACAGCACTGGTGATTA	463	2852−3314	
	CUV9R	CTGCAGCCCATGGTAGTAGG			
	CutaV-F4	CACTCCTACAAATAGCACAAGACAACAG	439	3264−3702	[3]
	CutaV-R3	TTGTTTCTGTCTCCCCATTGC			
	CUV10F	CACAAAGACACACAGGCAGC	385	3594−3978	
	CUV10R	TTTGGTGCCCAGTCAGTTTG			
	CuV 583 fw	TCAACAACTGAAGGMACCAGACTAAC	503	3833−4335	[1]
	CuV rev	TTCCATTGTCTTGGAGTGCG			
	CUV11F	TCCCAGAATCGTCACATATGGA	205	4252−4456	
	CUV11R	TTACAATGTGTAGTTTGGTAGACATC			

[a]Nucleotide position is based on the sequences of Cutavirus strain BR-337 and *RNaseP* from GenBank, accession number NC_039050 and NC_000010, respectively.

References

1. Väisänen E, Fu Y, Koskenmies S, Fyhrquist N, Wang Y, Keinonen A, Mäkisalo H, Väkevä L, Pitkänen S, Ranki A, Hedman K, Söderlund-Venermo M. Cutavirus DNA in malignant and nonmalignant skin of cutaneous T-cell lymphoma and organ transplant patients but not of healthy adults. Clin Infect Dis. 2019;68:1904–10.
2. Bhatia K, Goedert JJ, Modali R, Preiss L, Ayers LW. Merkel cell carcinoma subgroups by Merkel cell polyomavirus DNA relative abundance and oncogene expression. Int J Cancer. 2010;126:2240–6.
3. Phan TG, Dreno B, da Costa AC, Li L, Orlandi P, Deng X, Kapusinszky B, Siqueira J, Knol AC, Halary F, Dantal J, Alexander KA, Pesavento PA, Delwart E. A new protoparvovirus in human fecal samples and cutaneous T cell lymphomas (mycosis fungoides). Virology. 2016;496:299–305.
