# Supplementary material for: Cutavirus on the skin in an Asian cohort: identification of a novel geographically related genotype
Source: Virol J. 2023 Apr 17;20:69. doi: 10.1186/s12985-023-02029-8 (PMC10111705; doi:10.1186/s12985-023-02029-8)
Supplement: Supplementary file 2 — Additional file 2: figure S1 Phylogenetic analysis of cutavirus based on complete VP2 sequences. [file 12985_2023_2029_MOESM2_ESM.pdf]

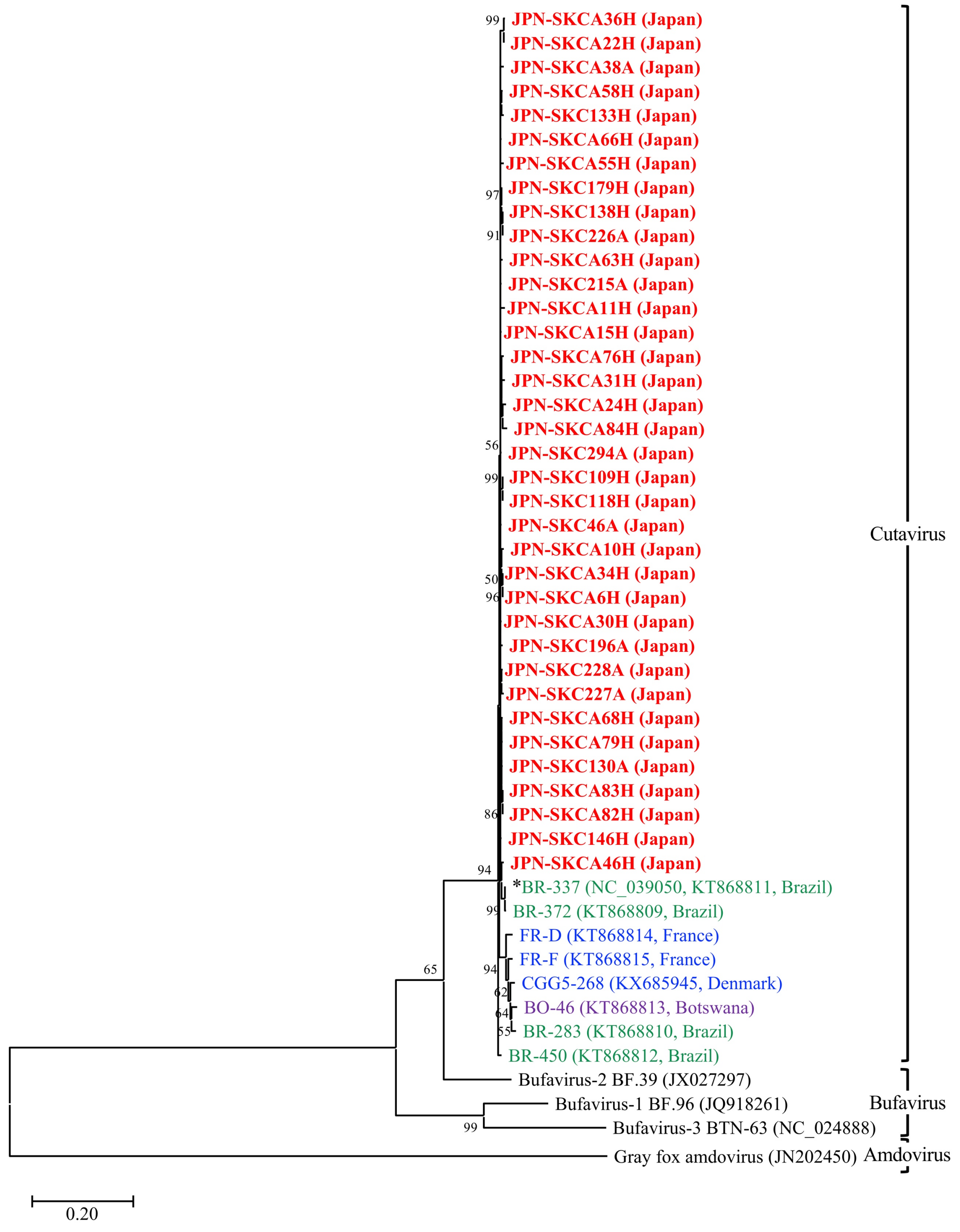

Figure S1 Phylogenetic analysis of cutavirus based on complete *VP2* sequences. Gray fox amdovirus and bufavirus were used as outgroups.
