# Supplementary material for: Cutavirus on the skin in an Asian cohort: identification of a novel geographically related genotype
Source: Virol J. 2023 Apr 17;20:69. doi: 10.1186/s12985-023-02029-8 (PMC10111705; doi:10.1186/s12985-023-02029-8)
Supplement: Supplementary file 3 — Additional file 3: table S2 Nucleotide identities between the near-full-length cutavirus sequences (4455 bp) identified in the current study. [file 12985_2023_2029_MOESM3_ESM.pdf]

Table S2 Nucleotide identities between the near-full-length cutavirus sequences (4455 bp) identified in the current study

	% Nucleotide identity																																			
Strain	JPN-SKCA6H	JPN-SKCA10H	JPN-SKCA11H	JPN-SKCA15H	JPN-SKCA22H	JPN-SKCA24H	JPN-SKCA30H	JPN-SKCA31H	JPN-SKCA34H	JPN-SKCA36H	JPN-SKCA38A	JPN-SKCA46H	JPN-SKCA55H	JPN-SKCA58H	JPN-SKCA63H	JPN-SKCA66H	JPN-SKCA68H	JPN-SKCA76H	JPN-SKCA79H	JPN-SKCA82H	JPN-SKCA83H	JPN-SKCA84H	JPN-SKC46A	JPN-SKC109H	JPN-SKC118H	JPN-SKC130A	JPN-SKC133H	JPN-SKC138H	JPN-SKC146H	JPN-SKC179H	JPN-SKC196A	JPN-SKC215A	JPN-SKC226A	JPN-SKC227A	JPN-SKC228A	JPN-SKC294A
JPN-SKCA6H	100																																			
JPN-SKCA10H	98.43	100																																		
JPN-SKCA11H	98.25	98.20	100																																	
JPN-SKCA15H	98.34	98.45	98.43	100																																
JPN-SKCA22H	98.09	98.11	98.16	98.27	100																															
JPN-SKCA24H	97.87	98.14	98.00	98.25	97.91	100																														
JPN-SKCA30H	98.61	98.29	98.97	98.50	98.29	98.18	100																													
JPN-SKCA31H	97.93	97.98	97.96	98.09	97.98	97.96	98.25	100																												
JPN-SKCA34H	99.73	98.50	98.29	98.38	98.16	97.93	98.65	97.98	100																											
JPN-SKCA36H	98.05	98.05	98.05	98.23	99.57	97.96	98.25	97.91	98.11	100																										
JPN-SKCA38A	97.78	97.76	97.53	97.96	97.71	97.60	97.76	97.67	97.80	97.67	100																									
JPN-SKCA46H	97.93	98.16	97.89	98.18	97.93	97.91	98.34	97.87	97.98	97.93	97.73	100																								
JPN-SKCA55H	98.88	98.34	98.20	98.41	98.11	98.07	98.54	97.91	98.74	98.07	97.82	97.98	100																							
JPN-SKCA58H	98.23	98.50	98.20	98.45	98.23	98.25	98.38	97.96	98.29	98.25	97.80	98.05	98.43	100																						
JPN-SKCA63H	98.25	98.72	98.11	98.47	98.11	97.98	98.25	97.96	98.25	98.07	97.76	98.00	98.29	98.29	100																					
JPN-SKCA66H	98.45	98.45	98.25	98.56	98.29	98.32	98.56	98.14	98.52	98.29	97.82	98.16	98.54	98.59	98.25	100																				
JPN-SKCA68H	98.27	98.34	98.07	98.36	97.93	98.14	98.38	98.16	98.36	97.89	97.94	98.23	98.18	98.18	98.23	98.27	100																			
JPN-SKCA76H	98.36	98.38	98.20	98.34	98.18	98.23	98.32	98.02	98.36	98.18	97.69	97.98	98.45	98.45	98.16	98.63	98.32	100																		
JPN-SKCA79H	98.32	98.54	98.34	98.65	98.20	98.18	98.50	98.25	98.38	98.16	97.78	98.20	98.32	98.38	98.52	98.41	98.52	98.41	100																	
JPN-SKCA82H	98.27	98.34	98.23	98.38	98.11	98.11	98.41	98.25	98.32	98.05	97.89	98.32	98.32	98.29	98.27	98.25	98.45	98.20	98.68	100																
JPN-SKCA83H	98.25	98.29	98.18	98.34	98.07	98.07	98.36	98.20	98.29	98.00	97.85	98.27	98.27	98.25	98.23	98.20	98.41	98.16	98.63	99.96	100															
JPN-SKCA84H	98.07	98.07	97.91	98.27	97.87	98.07	98.14	98.11	98.07	97.78	97.58	97.94	98.05	98.03	98.14	98.05	98.14	98.23	98.45	98.18	98.14	100														
JPN-SKC46A	98.29	98.43	98.16	98.38	98.14	98.05	98.14	98.00	98.23	98.05	97.55	98.23	98.34	98.32	98.45	98.29	98.09	98.20	98.36	98.29	98.25	98.00	100													
JPN-SKC109H	99.01	98.38	98.32	98.52	98.16	98.00	98.61	98.05	98.88	98.07	97.73	98.02	99.01	98.41	98.52	98.47	98.32	98.38	98.59	98.36	98.32	98.23	98.56	100												
JPN-SKC118H	98.99	98.36	98.29	98.50	98.14	97.98	98.59	98.02	98.86	98.05	97.71	98.00	98.99	98.38	98.50	98.45	98.29	98.36	98.56	98.34	98.29	98.20	98.54	99.98	100											
JPN-SKC130A	98.36	98.34	98.11	98.47	98.11	98.14	98.36	98.18	98.41	98.11	97.89	98.36	98.20	98.29	98.20	98.34	98.56	98.20	98.45	98.52	98.47	98.11	98.23	98.34	98.32	100										
JPN-SKC133H	98.38	98.32	98.27	98.43	98.16	98.00	98.50	98.07	98.47	98.09	97.87	98.09	98.41	98.50	98.23	98.43	98.45	98.27	98.47	98.32	98.27	97.96	98.25	98.38	98.36	98.38	100									
JPN-SKC138H	98.52	98.38	98.23	98.54	98.34	98.18	98.56	97.96	98.52	98.34	97.94	98.09	98.52	98.43	98.38	98.52	98.45	98.47	98.50	98.23	98.18	98.23	98.27	98.61	98.59	98.43	98.34	100								
JPN-SKC146H	98.47	98.65	98.25	98.52	98.20	98.14	98.41	98.11	98.56	98.20	97.96	98.29	98.43	98.52	98.50	98.45	98.45	98.45	98.59	98.45	98.41	98.11	98.34	98.41	98.38	98.45	98.45	98.59	100							
JPN-SKC179H	98.52	98.38	98.18	98.54	98.32	98.16	98.52	97.96	98.56	98.32	97.91	98.11	98.59	98.43	98.34	98.52	98.50	98.50	98.50	98.25	98.20	98.23	98.29	98.59	98.56	98.47	98.38	99.62	98.61	100						
JPN-SKC196A	98.16	97.93	97.89	98.14	97.80	97.96	98.14	97.91	98.20	97.78	98.00	97.73	98.14	98.00	97.93	98.11	98.32	98.20	98.25	98.18	98.14	97.96	97.82	98.14	98.11	98.20	98.11	98.18	98.14	98.25	100					
JPN-SKC215A	98.50	98.95	98.43	98.68	98.34	98.41	98.56	98.23	98.59	98.29	98.00	98.36	98.38	98.63	98.97	98.56	98.59	98.63	98.83	98.54	98.50	98.27	98.47	98.68	98.65	98.52	98.47	98.65	98.72	98.63	98.36	100				
JPN-SKC226A	98.45	98.32	98.16	98.54	98.25	98.09	98.47	97.91	98.52	98.25	97.82	98.05	98.47	98.34	98.29	98.47	98.41	98.41	98.41	98.16	98.11	98.16	98.20	98.52	98.50	98.38	98.27	99.66	98.54	99.75	98.09	98.61	100			
JPN-SKC227A	98.23	98.32	98.38	99.24	98.14	98.11	98.45	98.00	98.25	98.09	97.94	98.14	98.32	98.29	98.38	98.43	98.20	98.18	98.52	98.36	98.32	98.23	98.20	98.41	98.38	98.38	98.27	98.41	98.34	98.43	98.18	98.47	98.36	100		
JPN-SKC228A	98.38	98.38	98.29	98.83	98.25	98.18	98.54	98.09	98.41	98.20	97.85	98.09	98.36	98.43	98.34	98.79	98.18	98.36	98.47	98.38	98.34	98.18	98.29	98.47	98.45	98.34	98.34	98.43	98.54	98.41	98.09	98.56	98.34	98.99	100	
JPN-SKC294A	98.18	98.32	98.07	98.38	98.11	97.96	98.25	98.05	98.25	98.07	97.60	98.02	98.20	98.25	98.20	98.36	98.20	98.20	98.38	98.20	98.16	97.89	98.36	98.36	98.34	98.25	98.23	98.36	98.29	98.41	98.11	98.50	98.27	98.09	98.18	100
